# The Transactions of the American Medical Association

**Published:** 1852-02

**Authors:** 


*The Transactions of the American Medical Association. Instituted* 1847. Vol. iv. pp. 677.

The present volume of Transactions lacks none of the interest and merit which have distinguished its predecessors. It presents unequivocal evidence of the increasing vigor of the body from which it emanates; which, although yet in its infancy, is beginning to be felt through the whole medical profession of the United States. It is true, that on the great question of reform in the existing methods of medical education, the primary object of its organization, the Association has, as yet, made but little practical progress. It has, however, raised its standard and announced its wishes, and despite the efforts of the few who deem all such movements as chimerical or meddlesome, the great body of its members have steadfastly adhered to their original ground, and are determined to move forward until the united influence of the profession shall secure a more thorough system of education than has yet been realized.

The Report on Medical Education contained within the volume before us, affords ample evidence of the interest taken by the Committee in this branch of the business of the Association. It is an able and high-toned document, full and candid in its exposure of existing evils, and hopeful in its anticipations for the future. "Association," says the Report, "is the great means of creating, and bringing into action, a sound public sentiment amongst medical men." And again: "If all who desire that the standard of medical character and attainment be raised, would turn their influence into this channel, a great change would at once be effected in the condition of the profession of this country. The demi-quacks who so much disgrace our profession in the eyes of the public, would be driven from our ranks, public sentiment, both among medical men and in the community at large, would be renovated, and, consequently, the value of professional reputation would rise, while that of a merely popular one would fall, even as a source of emolument. A reform in our system of education would be rigorously prosecuted, and our schools would cease to send forth such numbers, as some often do, of unqualified physicians, to give currency to error, and delusion in medicine, to destroy health and life, and to bring contempt upon our noble science."

This testimony in favor of the value of association as the primary means of improving and elevating the profession, enforced as it is by many clear and cogent arguments, deserves the serious and renewed attention of medical men; and each one who becomes convinced of the simple proposition, that it is by this means that the desired reforms in medical education and character are to be obtained, has a plain course before him. Let him join the medical organization of his district; or if none exists, let him use his influence with his brethren to form one, which shall be governed by the regulations pointed out by the National or State Medical Society, of which it should form a component part. Let him not be a mere nominal or idle member of such a body; but turning aside from his personal duties and professional cares, let him devote a portion of his time and talents to give it character and efficiency.

In connection with this subject of medical organizations, we were gratified to notice that certain resolutions of the Philadelphia County Medical Society, proposing a modification in the present system of delegation to the National Association, were brought before the meeting at Charleston, by Dr. Yardley of this city, and received a respectful consideration, by being referred to a committee of five members with instructions to report next year.

The change proposed by the resolutions consists in so altering the constitution of the National Medical Association, as to make the delegates consist *only of persons appointed by the County and State Medical Societies.* It is obvious that by this arrangement a powerful motive would be created for the formation of these local Societies, as furnishing the only doors of entrance to the National Association, and hence, a more thorough and effective organization of the medical men of the country would be promoted.

If this be the main hope of the friends of medical progress, the grand lever whereby the incubus of ignorance and incompetency is to be removed, we can see no more effectual method of promoting it than by the adoption of the change recommended by the Philadelphia Society.

We repeat then, that the able report and resolutions on Medical Education, from the pen of Dr. W. Hooker, of Connecticut, together with the project for amending the constitution to which we here refer, afford gratifying evidence of a progressive movement in the right direction, and we think that those most skeptical of the advantages of association, must admit that there is at least an earnestness and uniformity in the action of this great national body, which, whatever may be its future influence on the interests of the medical profession, must command the respect and deference of every member of it, who is alive to the true interests of his calling.

Another important feature in the proceedings of the last meeting, is the change effected in the mode of bringing medical papers before the Association. The original plan of presenting elaborate reports on the progress of the different branches of medical science during the year, has been superseded by the appointment of Committees to consider and report upon special subjects of inquiry, selected as most suitable for investigation. The selection for the coming year embraces twenty-seven subjects, most of which possess high interest to the medical enquirer. Should these committees fulfil their duty in a creditable manner, a vast fund of useful information will be thrown before the profession, upon a variety of important topics, while the character of the Association as a scientific body will be greatly enhanced, both at home and abroad.

As an additional means of securing valuable contributions, it was agreed to invite volunteer papers upon any subject which their authors may choose, to be presented to the Association at the discretion of a committee appointed to receive them and to judge of their merits; and, in order to stimulate talent by competition, a prize of fifty dollars, or a gold medal of that value, will be awarded to the authors of each of the five papers presented to the Association, which the committee shall consider the most meritorious.

These measures we cannot but esteem as highly important, in imparting to the Association a new element of strength and usefulness, by attracting towards it cultivated minds, and making it the channel through which copious streams of knowledge will flow. We believe there are many young men in our profession, gifted with a high order of talent, who suffer their minds to languish and grow dull, for want of a stimulus to exertion, such as is here held out. By entering the list of competitors for the honorable position now offered to their acceptance, latent powers will be developed, and new energies will be aroused which may lead to success, and exert a powerful influence on the future life of the aspirant. Already has the successful candidate for the first prize essay presented to the Association, obtained a position amongst his compeers, which years of studious toil might not have secured him, had not his labors been directed into this particular channel.

Another matter which appears to have claimed the attention of the Association, was the subject of demonstrative midwifery. This was specially referred to the committee on medical education by the last meeting; and has received from it a full, candid, and deliberate examination. The conclusion of the committee is against the plan adopted by the Institution which introduced the subject to the notice of the Association.

"Granting," says the Committee, "all that can be claimed with any plausibility for the advantages mentioned, they are not of sufficient value to make it proper, that woman, in the hour of her extremity, should be made the subject of public exhibition."

This report was deemed of sufficient importance to call for the passage of a resolution confirming the opinions thus advanced, and, so far as its unanimous approval by the Association is an index of the general sentiment of the profession, it is clear and decided.

One other matter in the minutes attracted our attention, and though not of great importance, we feel constrained briefly to call the attention of our readers to it. It is a resolution of Dr. A. H. Stevens, of New York, for the appointment of a committee to procure a tablet with a suitable inscription, commemorative of the Charleston meeting, and in order "signalize the gratitude of the members for the extraordinary professional and social enjoyment that accompanied it."

Now no one can doubt that our medical brethren in Charleston did everything which kindness and hospitality could suggest, to render the visit of the members of the Association to their city agreeable and even memorable; but in this respect, they did no more than their feelings prompted, or than the profession in other cities have done, and may do hereafter. It was, therefore, unnecessary, and may even be deemed invidious, to signalize their kindness by extraordinary proceedings, and we cannot but regret that the Association departed from its accustomed course in so doing.

The reports of the standing and special committees cover 500 pages of the volume before us. They embrace the usual amount of matter of scientific interest, and are drawn up with decided ability.

It would be impossible, within our limits, to analyze the vast amount of material thus presented, and we shall attempt no more than a brief notice of such points as seem to possess extraordinary interest.

The report on "Practical Medicine" from the pen of Dr. Austin Flint, of Buffalo, is especially interesting, from its containing a history of several remarkable epidemics, which have prevailed in various sections of the Union during the year, such as cholera, epidemic dysentery, typhoid fever, dengue, &c. It is true, that these reports have reference to a few only of the epidemic diseases of the year, and those prevailing in limited sections; yet, as contributions to the meagre fund of knowledge which we possess on this important branch of medicine, they must be highly prized:

"Epidemic cholera, (says the report,) has prevailed to a considerable extent within the limits of the United States during the past year, and, as heretofore, its progress has been attended by a large fatality. Its ravages have been confined chiefly to places situated on or near the Mississippi and Ohio Rivers, the north-western lakes, more especially the two former, and to California. In the States situated on the Atlantic coast, and between the latter and the Alleghany Mountains, it does not appear that the disease has prevailed as an epidemic.

Prof. Alanson Clark, of the N. Y. College of Physicians and Surgeons, in a letter to the chairman of the committee, states that, on inquiring of numerous practitioners of the city of New York if they had met with the disease, the answer was uniformly that they had not, nor had they heard of a single case.

He adds that, on applying at the city inspector's office, he found seven deaths had been reported as caused by cholera, one of these being in

June, and six in July; but of the correctness of the diagnosis in these cases he is not fully assured.

"At Boston, the occurrence of a single case only has been chronicled during the year.

"No reports of cases occurring at Philadelphia, Baltimore or other places within the territory stated to have been exempt from the epidemic, have fallen under notice. Reports more or less full, in medical journals, and communications addressed to the committee, furnish historical accounts of the disease as it has prevailed at New Orleans (La.), St. Louis (Mo.), Louisville (Ky.), Cincinnati (Ohio), Columbus (Ohio), Keokuk and Burlington (Iowa), Kalamazoo (Mich.), Chicago, (Ill.), Buffalo (N. Y.), and Sacramento (California). The committee will notice the more important facts contained in the several reports, and then offer such remarks as may suggest themselves."

A succinct, and in some cases, highly interesting history of the disease as it prevailed in different localities, follows, and the result is thus summed up by the Committee:

"The accounts that have thus been submitted are far from affording a complete representation of the progress of cholera during the past year. They relate to the epidemic only as it existed at several prominent places, and they are, in general, defective in not embracing various particulars of importance in the history of the disease. Places on the great western rivers and lakes, from which no authentic reports have been made, have suffered more or less. The disease is also known to have prevailed to a considerable extent on the vessels navigating these waters.

"The majority of places from which accounts have been presented, are connected with each other by a continuous water communication, by means of which an extensive commercial intercourse is kept up, forming, also, a thoroughfare for a large proportion of the immense tide of foreign and domestic immigration. This fact, probably, in the minds of some persons, will give rise to an inquiry respecting the transportation of the disease successively to these places. With reference to this inquiry, it is to be observed that, as it would seem, to New Orleans belongs priority in the presence of the disease, it having been at no time absent from that city, although never prevailing to a great extent, and in a great measure confined to foreign immigrants. At Louisville, it appeared in two successive short outbreaks in June and July, the determining causes being traceable to local sources. At Cincinnati, it prevailed from June to the 15th of August. At Chicago, from the 23d of June to September 1st. At Buffalo, it can hardly be said to have prevailed as an epidemic, but the greater number of cases occurred in July, August and September. With reference to the present inquiry, it is much to be regretted that accurate reports have not been made of the occurrence of the disease at numerous places on the same extensive routes of water communication. The fact that the disease was in a great measure limited to these routes, appearing in but few other portions of the United States, is worthy of note in this connection. It appears that in all the places that have been mentioned, in which statements relative to the point are made, the development of the disease was preceded by cases occurring among persons lately arrived from other places in which the disease existed. The uniformity of this fact invests it with a certain degree of significance; but, it is to be observed that these cases frequently preceded for a considerable length of time, nor did the subsequent indigenous cases have any apparent connection with them. Although, as just stated, the epidemic is generally preceded by imported cases, the converse is not true, viz., that the importation of cases is uniformly followed by indigenous cases. The history of the epidemic, moreover, in most of the places mentioned in this report, shows, not only a commencement at a period more or less remote and disconnected from the introduction of the disease, but a career which seems necessarily to involve a prevailing epidemic influence having a limited duration, which we should suppose would be otherwise if each case contributed, in any manner, to the special cause. In view of these considerations, while it must be admitted that the transportation of cases from one place to another apparently has some connection with the subsequent development of the disease in the latter, the nature and extent of this relation cannot, in the present state of science, be defined or explained."

As an appendix to this section of the report, is an admirable paper by Dr. George Mendenhall, entitled, "An account of Cholera as it appeared in Cincinnati during the year 1850," and a "Report on Fevers, &c., of New Orleans, by Dr. E. D. Fenner," which contains a history of epidemic cholera as it appeared in that city.

Epidemic dysentery has appeared in some sections of the United States, during the year, but the extent of its prevalence, and the peculiar circumstances attending its origin, could not be ascertained by the few facts presented to the Committee. A point of great interest developed by the reports received on this subject, is the account given by different contributors of the treatment found most successful in their hands. The conclusions to be drawn from these are thus announced by the Committee:

"Considering the great diversities of character which an epidemic disease is apt to assume in different sections of country and seasons—diversities from which dysentery is certainly not more exempt than other epidemics—the practical views of a pretty large number of observers, which are presented in the foregoing collection, exhibit, in some important features, a uniformity which is worthy of note. It is to be considered, moreover, in this view of the subject, that the different observers, by whom these practical views are contributed, are not only remotely situated with respect to each other, but there is no reason to suppose that the several communications had any connection with each other, by way of suggestion, or otherwise.

"In so far as the facts and opinions embraced in these communications are to be considered as supplying data for an induction, the several conclusions may be summed up as follows:

"*a.* In the management of epidemic dysentery, as it has prevailed at the places and periods referred to in the papers that have been contributed on the subject during the past year, depletion by blood-letting, cathartics, and the free use of mercurials, are not required, but will be likely to do harm.

"*b.* Opium, exhibited in decided doses, exerts a salutary influence in relieving painful symptoms, and controlling the severity of the disease.

"*c.* The nitrate of silver is a valuable remedy, employed with a view to a local and general effect.

"*d.* Tannic acid, and the acetate of lead, under circumstances denoting the propriety of astringents, are useful remedies.

"*e.* To support the powers of the system by a timely resort to tonics, stimulants and alimentation, is an important end to be kept in view in the treatment.

"These propositions embody therapeutical principles by no means entirely new, and it is difficult to ascertain to what extent they are in conformity with prevailing opinions. The committee, therefore, are unable to say in how far they are to be considered in the light of an improvement in the management of the affection to which they relate."

An epidemic fever of a singular character, prevailed during the year in many of the Southern States, under the name of Dengue, or "break-bone fever." An excellent descriptive history of this singular epidemic is contained in the report before us, the main features of which we would gladly transfer to our pages, did not our limited space forbid. We can only refer to the sources from which the materials for this history were derived, in order that any of our readers, who may feel inclined, can examine for themselves, viz: "A history of the Epidemic Dengue, as it prevailed in Charleston in the Summer of 1850," by Prof. Samuel Henry Dickson, of the Medical College of South Carolina, published in the Charleston Medical Journal and Review, November, 1850. "History of the Break-bone fever, an epidemic which prevailed in Charleston in the Summer of 1850, by Wm. T. Wragg, M. D.," same Journal, March, 1851. "History and treatment of the Dengue fever, prevailing in Augusta, in the year 1850, by Henry F. Campbell, M. D.," Southern Medical and Surgical Journal, Jan. 1851.

The Report on Surgery, by Dr. Paul F. Eve, opens with a brief description of the value of anæsthetic agents, in which the Committee express an "undiminished confidence in them, and a grateful admiration of the blessings they have conferred upon mankind."

Several cases of cure of Aneurism by compression, are reported amongst the events of the past year, with many other cases and operations of interest, furnished to the Committee by numerous contributors.

The report is accompanied by several elaborate and valuable surgical papers. Amongst them, Dr. Washington L. Atlee's table of all the known operations for Ovariotomy, from 1701 to 1851, comprising 222 cases, with a synoptical history of each case; and the surgical statistics of the New York Hospital, compiled from the hospital records by Frederick D. Lente, M. D., are especially worthy of notice.

The results of the operation in the 222 cases of ovariotomy, collected by Dr. Atlee, are thus summed up:

"I. Of these 222 cases, 52 were of the minor section, 153 of the major, and 17 unknown.

"II. Of the 52 minor operations, 39 recovered, and 13 died, or 1 in every 4, or 25 cases in 100.

"III. Of the 153 major operations, 95 recovered, and 58 died, or 1 in every $2\frac{37}{58}$, or 37.91 cases in 100.

"IV. Of the 17 unknown sections, 12 recovered, and 5 died, or 1 in every $3\frac{2}{5}$, or 29.41 cases in 100.

"V. Of the 222 cases, 146 recovered, and 76 died, or 1 in every $2\frac{35}{38}$, or 33.78 cases in 100.

"VI. Of the 222 cases, 57 were not completed, or 1 in every 3 17-19ths, or 25.68 cases in 100.

"VII. Of the 222 cases, there was no tumor in 6, or 1 in every 37, or 2.7 cases in 100.

"VIII. Of the 57 unfinished operations, 24 were the large section, 27 the small, and 6 unknown.

"IX. Of the 24 unfinished large sections, 17 recovered, and 7 died, or 1 in every 3 3-7ths, or 29.17 cases in 100. The proportion of unfinished operations in the large sections, is as 24 to 153, or 15.69 cases in 100.

"X. Of the 27 unfinished minor sections, 23 recovered, and 4 died, or 1 in every $6\frac{3}{4}$, or 14.81 cases in 100. The proportion of unfinished operations in the small sections, is as 27 to 52, or 51.92 cases in 100.

"XI. Of the 6 unfinished unknown sections, 5 recovered and 1 died, or 1 in every 6, or $16\frac{2}{3}$ cases in 100.

"XII. Of the 57 unfinished operations, 45 recovered and 12 died, or 1 in every $4\frac{3}{4}$ cases, or 21.05 cases in 100.

"XIII. Of the 57 unfinished operations, 25 were merely exploratory, all of which recovered.

"XIV. Of the 6 operations in which no tumor was found, 5 were major and 1 minor; 3 of the former recovered, 2 died; and the minor recovered; making 4 recoveries, 2 deaths, or 1 in every 3."

The rate of mortality after the operation according to the abôve table, is $26\frac{1}{2}$ per cent.

The Report of the Committee on Obstetrics, by Dr. D. Humphreys Storer, covers over fifty pages of the Transactions, and contains a full resumé of the facts and interesting or important cases, in this department, which have been brought to the knowledge of the committee, through the medium of the Medical Journals published during the year. It is a valuable annual abstract of this branch of medicine, and may be consulted with advantage by the practitioner.

We have already referred to several important matters discussed in Dr. Hooker's admirable Report on Medical Education, and might, with advantage, present to our readers a full analysis of it under its appropriate head; but our limits forbid us to do more than to commend its careful perusal to the members of the profession generally, believing, as we do, that it contains a lucid and forcible argument of the necessity of reform in our system of medical education, together with the true principles upon which such a reform should be based. We were gratified in observing amongst the proceedings of the Association, the following resolution in reference to this report, which we cordially recommend to the bodies to which it refers: "Resolved, That it be recommended to the several State Medical Societies throughout the Union, to procure a re-publication of the Report of the Committee on Medical Education, for general distribution among the profession."

This recommendation is now the more imperative from the fact that, owing to a late calamitous fire in Philadelphia, the larger part of the edition of the present volume was consumed, leaving but a little over 500 hundred copies for distribution; thus cutting off the great body of the profession from the reading of this and the other reports.

The report on Medical Literature, Dr. Thomas Reyburn,

Chairman, contains a full and satisfactory review of the periodical medical literature of the year, with some excellent observations on the general tone and character of the productions which appear in the Medical Journals, and the means calculated to improve them. The Committee complain of the indisposition of Editors to contribute to their own pages, and of their want of care in correcting the contributions of their correspondents. "More of original contributory matter," says the Committee, "and strict supervision of their columns on the part of the Editors, would, we are convinced, improve the character of this important department of our literature, and are most certainly portions of editorial duty due to readers, by virtue of implied contract with them."

One section of this report is occupied with an able and ingenious disquisition upon the causes and circumstances which are impeding the growth of American medical literature, and which make it so inferior to the European, together with suggestions as to the best means of fostering and improving it.

The committee attribute much to the establishment and influence of scientific societies of a high grade, as a means of arousing an impulse to literary effort. The following extract upon this point is worthy of attention:

"The organization of the National Medical Association has aroused a feeling of interest, and awakened a spirit of inquiry into the state of the profession, which need only to be taken advantage of, for the furtherance of the end we have in view. Men from the far west, the borders of civilization, have left their weary round of toil, toil so weary that one might fancy it to engender hatred of the profession which exacts so much for the bread of life, to gather to its councils; the wealthy southerner has roused from his reputed indolence, and journeyed even to the distant north to glean some lessons in professional wisdom; and will it be said that, where such sacrifices are made through love of profession, greater, far greater, will not be made through the laudable ambition of identifying themselves with that profession in after years, of giving to it some record of their knowledge or experience, which will register their names upon its annals?

"It is no novelty advanced that the successful practitioner is by no means always the most scientific light in his profession. We all know and feel, unfortunately, that manner, more than matter, wins the human heart; and we all know, from records of others, even if we have not seen such, that many a gem in our profession drags on life wearily, from want of encouragement from public favor, whilst many a shallow pretender palms off charlatanry for professional skill, and rides prosperously on the breath of public applause through mere suavity of manner and polish of address. That this ever has been, and ever will be the case, none can doubt. But it is equally certain that, in Europe, merit in dire indigence, and talent in obscure poverty, are rescued from oblivion by the recognition of its labors by scientific bodies, the ability and justice of whose members are a sufficient guarantee to pass current the coin they stamp. Many a name, before which the practitioner in America might pause with a feeling of veneration, almost of envy, would have gone down to the grave unhonored and unknown, crushed by the bitter struggle with poverty, had not a medal or an honorary membership, aroused first friends, then neighbors, and, lastly, the reluctant, fawning world, to appreciation of merit which spoke not for itself; and many a name even now familiar to our ears as noble in our profession, has yet no light to cheer on labor, has earned as yet little reward for toil, save the recommendations to public favor of which we speak. These have ever been found powerful impulses to exertion, and we will not believe that in our country they would prove less able incentives."

With these views the Committee regard with favor the formation of a society, in connection with the National Medical Association, "for the reception and discussion of original scientific papers;" a measure which is partially accomplished in another way, by the changes adopted in the organization of the committees, to which we have already referred.

A highly interesting report on Hygiene, from the pen of Dr. Gailliard, of Charleston, completes the series. This report is confined to the following special subjects of inquiry, viz:

First, The best methods of warming and ventilating public and private buildings.

Secondly, The importance of collecting, in connection with our system of meteorological records, information in regard to the diseases of the different parts of the country; a subject presented to the notice of the Association by the Smithsonian Institute.

Thirdly, The consideration of the following Preamble and Resolution:

"Whereas, The clerical profession often, though perhaps sometimes unwarily, yield their extensive influence in the community to give currency to quackery and quack medicines, therefore,

Resolved, That this subject be referred to the Committee on Hygiene, to consider and report at the next meeting of the Association."

The importance of the subjects discussed in this report, and the ability with which they are handled, cause us to regret that so few of the members of the profession will have an opportunity of perusing them. We would, therefore, hope either that one or more of the Medical Journals would publish the report in full, or that local Medical Societies would print editions for extensive circulation.

The volume closes with the Prize Essay, which is an elaborate and valuable paper, "On the Corpus Luteum of Menstruation and Pregnancy," by John C. Dalton, Jr., M. D.

This essay is embellished with a series of highly finished colored plates, illustrative of the subject, introduced at considerable expense, and highly creditable to American art. It is, we believe, universally regarded as a rich original contribution to Physiology, evincing on the part of the author a high order of talent, and greatly enhancing the value of the present volume of Transactions.

The essay covers about 100 pages, but as it will be issued in a separate volume, and from its character would seem to demand from us a distinct notice, we shall forbear even a statement of its contents at this time. P.

